# Rac-Induced Left Ventricular Dilation in Thyroxin-Treated ZmRacD Transgenic Mice: Role of Cardiomyocyte Apoptosis and Myocardial Fibrosis

**DOI:** 10.1371/journal.pone.0042500

**Published:** 2012-08-24

**Authors:** Mohammad T. Elnakish, Mohamed D. H. Hassona, Mazin A. Alhaj, Leni Moldovan, Paul M. L. Janssen, Mahmood Khan, Hamdy H. Hassanain

**Affiliations:** 1 Department of Anesthesiology, and Dorothy M. Davis Heart and Lung Research Institute, The Ohio State University, Columbus, Ohio, United States of America; 2 Department of Physiology and Cell Biology, and Dorothy M. Davis Heart and Lung Research Institute, The Ohio State University, Columbus, Ohio, United States of America; 3 Department of Pulmonary, Allergy, Critical Care and Sleep Medicine, and Dorothy M. Davis Heart and Lung Research Institute, The Ohio State University, Columbus, Ohio, United States of America; 4 Department of Internal Medicine, and Dorothy M. Davis Heart and Lung Research Institute, The Ohio State University, Columbus, Ohio, United States of America; University of Western Ontario, Canada

## Abstract

The pathways inducing the critical transition from compensated hypertrophy to cardiac dilation and failure remain poorly understood. The goal of our study is to determine the role of Rac-induced signaling in this transition process. Our previous results showed that Thyroxin (T4) treatment resulted in increased myocardial Rac expression in wild-type mice and a higher level of expression in Zea maize RacD (ZmRacD) transgenic mice. Our current results showed that T4 treatment induced physiologic cardiac hypertrophy in wild-type mice, as demonstrated by echocardiography and histopathology analyses. This was associated with significant increases in myocardial Rac-GTP, superoxide and ERK1/2 activities. Conversely, echocardiography and histopathology analyses showed that T4 treatment induced dilated cardiomyopathy along with compensatory cardiac hypertrophy in ZmRacD mice. These were linked with further increases in myocardial Rac-GTP, superoxide and ERK1/2 activities. Additionally, there were significant increases in caspase-8 expression and caspase-3 activity. However, there was a significant decrease in p38-MAPK activity. Interestingly, inhibition of myocardial Rac-GTP activity and superoxide generation with pravastatin and carvedilol, respectively, attenuated all functional, structural, and molecular changes associated with the T4-induced cardiomyopathy in ZmRacD mice except the compensatory cardiac hypertrophy. Taken together, T4-induced ZmRacD is a novel mouse model of dilated cardiomyopathy that shares many characteristics with the human disease phenotype. To our knowledge, this is the first study to show graded Rac-mediated O_2_·^−^ results in cardiac phenotype shift *in-vivo*. Moreover, Rac-mediated O_2_·^−^ generation, cardiomyocyte apoptosis, and myocardial fibrosis seem to play a pivotal role in the transition from cardiac hypertrophy to cardiac dilation and failure. Targeting Rac signaling could represent valuable therapeutic strategy not only in saving the failing myocardium but also to prevent this transition process.

## Introduction

Cardiovascular diseases remain the main cause of death in the Western world, with heart failure representing the highest increasing subclass over the past decade [1]. Heart failure is classically induced by several common disease stimuli, such as hypertension, myocardial infarction or ischemic coronary artery diseases [2], [3]. Most of these stimuli initially provoke a stage of cardiac hypertrophy [4]. Hypertrophy can be a compensatory response to enhance contractility and preserve cardiac output exclusive of undesirable pathology. Nevertheless, persistent stress can progress this compensatory process into a decompensated state with reflective alterations in gene expression profile, contractile dysfunction, and extracellular remodeling [5], [6]. Pathological signs in end-stage heart failure share various common features apart from the causal etiologies, such as ventricular wall thinning, chamber dilation, cardiomyocyte loss, and severely increased interstitial fibrosis [7], signifying that intracellular signaling pathways triggered by different stressors may congregate to a number of common targets [8]. As highly conserved signaling pathway, the Rho/Rac signaling may be a common mediator in these pathological remodeling processes.

Previous studies using isolated cardiomyocytes from transgenic mice over-expressing Rac1 in the myocardium showed the importance of Rac1 in the development of cardiac hypertrophy [9]. Also, over-expression of a constitutively active form of human Rac1 in the hearts of transgenic mice resulted in cardiac hypertrophy and cardiac dilation [10]. Additionally, It has been reported that in human heart failure, increased NADPH oxidase–dependent ROS production is coupled with increased membrane expression and activity of Rac1 [11]. More recently, we have shown for the first time the conservation of Rho/Rac proteins in both plant and animal kingdoms *in-vivo.* Furthermore, we showed that over-expression of a constitutively active cardiac-specific form of ZmRacD gene in the transgenic mice resulted in cardiac hypertrophy as well as a moderate decrease in systolic function in older mice. Besides, the activation of ZmRacD expression with T4 for two weeks led to cardiac dilation and severe systolic dysfunction in adult transgenic mice. However, the same T4 treatment in wild-type mice resulted in a lower increase in myocardial expression of endogenous Rac with preserved cardiac function and left ventricular (LV) internal diameters [12]. Moreover, our current data shows that this preserved cardiac function in wild-type mice after T4 treatment is linked to physiologic cardiac hypertrophy.

While physiological versus pathological hypertrophy can be obviously distinguished by numerous qualitative and quantitative parameters, the basic mechanisms and their interrelationship remain divisive. Most notably, the signaling mechanisms inducing the critical transition from compensated hypertrophy to decompensated heart failure remain poorly understood [6], [13]. In the current study we hypothesize that recognizing the functional, structural, and molecular differences between these two distinct phenotypes; the Rac- induced physiologic cardiac hypertrophy in wild-type mice and pathologic cardiac dilation in ZmRacD transgenic mice after T4 treatment, will provide better understanding of the molecular mechanisms behind these diseases. This will allow for better treatment options in the future.

## Methods

### Animals

Mice were bred and maintained at the W.M. Keck Genetic Research Facility of The Ohio State University (OSU), and all experimental procedures and protocols used in this study were approved by the Animal Care and Use Committee of OSU, conforming with the guide for care and use of laboratory animals published by U.S. National Institutes of Health (NIH publication No. 85-23, revised 1996). 6-to-8-months-old sex-matched heterozygous ZmRacD transgenic mice and nontransgenic wild-type littermates were used in this study. Unless otherwise stated 6 to 8 mice per genotype were used for each experiment.

### Thyroxin (T4) and Drug Treatments

T4 was prepared as described by Pierres and Gaugain-Hamidi [14] with slight modification to increase both the solubility and stability of T4 preparation. Briefly, each 100 ml of the preparation contained all of the following: (Sodium-L-thyroxin: 2 mg, NaHCO_3_: 0.336 g, hydroxypropyl-β-cyclodextrin: 2 g, EDTA: 0.1 g, ammonium chloride: 0.5 g, ethanol: 15 ml, all obtained from Sigma), *pH* was adjusted to 8.5, then completed to 100 ml with double distilled water and stored at 4 C°.

Animals were divided into 6 groups based on genetic background and treatment (n = 8/group) as follows: wild-type; wild-type + T4; ZmRacD; ZmRacD + T4; ZmRacD + carvedilol + T4; ZmRacD + pravastatin + T4. The study duration was extended to 3 weeks instead of 2 weeks [12] based on previous studies demonstrating that the largest part (70–80%) of the myocardial remodeling process in rodent e.g. rat occurs within the first 3 weeks [15]. Basal echocardiography parameters were determined before starting T4 treatment and on a weekly basis thereafter, till the experiment was terminated. T4 (200 µg/Kg) was injected intraperitoneally as previously described [12].

Carvedilol (2 mg/Kg) or pravastatin (10 mg/Kg) were administered by intraperitoneal injection 1 hour before T4 administration. Carvedilol was prepared as reported in [16] with some modifications. Briefly, carvedilol from Sigma was dissolved in a minimal amount of DMSO, and then diluted with 5% dextrose solution containing 1–2 drops of glacial acetic acid (final DMSO concentration ≤0.1%). The dose of carvedilol used in this study was based on previous studies that showed its effectiveness in a rat model of cardiac dilation and it is roughly equivalent to the carvedilol dose in human [17]. Pravastatin was prepared and injected as previously described [12]. At the end of the treatment period animals underwent echocardiography. Directly after echocardiography, animals were sacrificed, and hearts were excised and processed for further experiments.

### Echocardiography

*In- vivo* cardiac dimension and contractile function in both wild-type and transgenic mice were evaluated using a high-frequency ultrasound imaging system (VEVO 2100, Visual Sonics, Toronto, ON, Canada). Experimental mice were initially anesthetized with isoflurane at a concentration of 2% and thereafter maintained at 1% isoflurane using nasal prongs during the whole procedure. The measurements were taken from the parasternal short-axis view in M-mode to view the LV movement during diastole and systole corresponding to the electrocardiogram. All data and imaging were analyzed by the Visual Sonics Cardiac Measurements Package.

### Myocardial Rac-GTP Activity

Rac-GTP activity was detected using a new commercially available antibody that has been reported to be specific for the active GTP-bound state of Rac1 (Neweast Biosciences, cat. #: 26903). This antibody has been recently characterized as a probe to examine Rac1 activation status in formalin-fixed paraffin embedded (FFPE) samples [18]. Immunohistochemical staining of Rac1-GTP was performed on 4-µm sections from FFPE mid-ventricles. Antigen retrieval was performed by a heat method in which the specimens were placed in Dako's target retrieval solution, pH 6.1 (Dako code S1699) for 25 minutes at 95°C using a steamer and cooled for 15 minutes in solution. Slides were then placed on a Dako Autostainer, immunostaining system. To minimize nonspecific staining, the Animal Research Kit from Dako (code K3955) was used according to the package insert. Primary antibody was diluted 1∶150. Slides were initially blocked for endogenous biotin, and then the biotinylated primary antibody was added for 1 hour, followed by the labeled streptavidin for 30 minutes. Finally, the slides were developed with diaminobenzidine (DAB) chromogen for 10 minutes, lightly counterstained in Richard Allen hematoxylin, dehydrated through graded ethanol solutions and coverslipped. 3 to 5-images per heart were acquired with a 20× objective for morphometric evaluation of the brown-stained Rac-GTP using MetaMorph image analysis software 7.1.2.0 (Molecular Devices, CA).

### Myocardial Superoxide (O_2_·^−^) Production

*In-situ* production of myocardial O_2_·^−^ was assessed by the fluorescent dye, dihydroethidium (DHE) as described previously [12]. Briefly, hearts from both wild-type and transgenic mice were placed immediately in ice-cold PBS, washed and then embedded in OCT for cryosectioning. Frozen sections (6-µm) from the hearts were incubated with DHE (10 µM; Sigma-Aldrich) for 30 min at 37°C in a dark chamber. After rinsing with PBS, sections were mounted in aqueous medium (Gel Mount, Sigma) and 3 to 5-images per heart were acquired with a 40× objective using a fluorescence microscope (Nikon TE 300, Tokyo, Japan). The red fluorescence intensity was determined using the MetaMorph image analysis software 7.1.2.0.

### Cardiomyocyte Length and Cross Sectional Area

4-µm FFPE mid-ventricles sections were lightly stained with Masson's trichrome. 3 to 5-images per heart were acquired with a 40× objective with a Nikon Eclipse TS-100F inverted microscope equipped with a Lumenera Infinity 1-2C camera (Nikon Instruments Inc., Melville, NY). The length and cross sectional area of cardiomyocytes were measured using the MetaMorph image analysis software 7.1.2.0 as previously described [12]. An average of 50–150 cells/group have been evaluated for cardiomyocyte length and 200–500 cells/group have been evaluated for cardiomyocyte cross sectional area.

### Myocardial Fibrosis

Standard Masson's trichrome staining was performed on 6-µm sections from FFPE mid-ventricles. 3 to 5-images per heart were acquired with a 40× objective for morphometric evaluation using MetaMorph image analysis software 7.1.2.0 and collagen deposition areas in both ventricles were calculated from different animals.

### Western Blot Analysis

Western blots were performed to determine the relative cardiac expression of the extracellular signal-regulated kinase (ERK1/2), p-ERK1/2, jun NH2-terminal kinase (JNK), p-JNK, p38 MAPK, p-p38 MAPK, caspase-8, cleaved caspase-3 and tumor necrosis factor-α (TNF-α) in both wild-type and transgenic mice as described in our previous reports [19]. Membranes were incubated with specific antibody for p-ERK1/2 and p38 MAPK (1∶1000; Cell Signaling), ERK1/2, JNK and p-JNK (1∶500; Santa Cruz), p-p38 MAPK (1∶500; Cell Signaling), or caspase-8, cleaved caspase-3 and TNF-α (1∶250; Cell Signaling). Secondary antibodies were goat anti-rabbit and anti-mouse IgG-HRP (1∶2000; Santa Cruz). Antibody signals were detected by an enhanced chemiluminescence kit (Pierce, USA), and GAPDH (1∶3000; Cell Signaling) was used as an internal control for equal protein loading. All Western blot results are expressed as the ratio of respective proteins to GAPDH.

### Myocardial Cell Apoptosis

To detect fragmented DNA, *in-situ* terminal deoxynucleotidyl transferase-mediated nick-end labeling (TUNEL) assay was performed as specified in the *in-situ* apoptosis detection kit instructions (Roche, cat. # 11 684 817 910) using 4-µm FFPE mid-ventricles sections. The slides were developed with a DAB chromogen for 10 minutes. Slides were then lightly counterstained in Richard Allen hematoxylin, dehydrated through graded ethanol solutions and coverslipped. 3 to 5-images per heart were acquired with a 40× objective for morphometric evaluation of the brown-stained apoptotic nuclei using MetaMorph image analysis software 7.1.2.0. Data are expressed as the percentage of apoptotic nuclei relative to normal nuclei.

### Data Analysis

Data were analyzed by ANOVA followed by Tukey-Kramer Multiple Comparisons post-hoc test, and are presented as means ± SEM. A two-tailed value of P<0.05 was considered statistically significant.

## Results

### T4-Stimulated Rac Expression is Associated with Increased Myocardial Rac-GTP Activity and Superoxide (O2·^−^) Production

**Figure 1:**
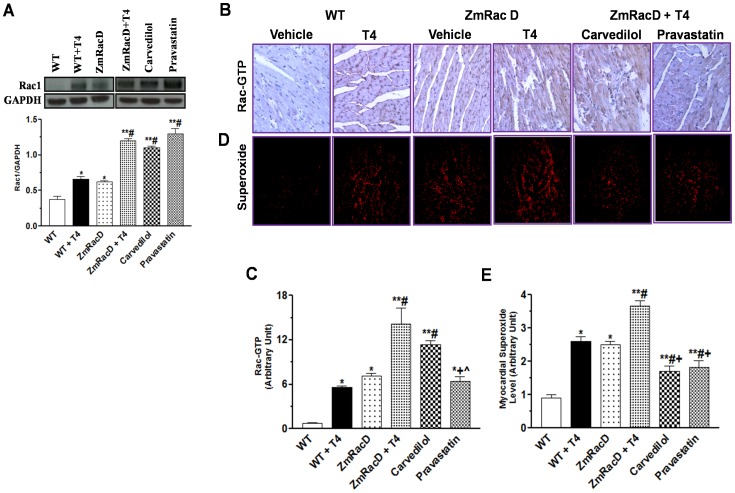
T4-Stimulated Rac Expression is Associated with Increased Myocardial Rac-GTP Activity and Superoxide (O2·^−^) Production. Western blot analyses of myocardial Rac1 (**A**) Representative images of Rac-GTP immunostaining in FFPE mid-ventricle sections, with bar graph for the mean intensity of the brown colored active Rac-GTP in arbitrary units (**B & C**). Representative images of myocardial O_2_^·^−^^ generation with DHE in frozen sections of mice hearts, with bar graphs for mean fluorescence intensity in arbitrary units (**D & E**). * is significant change compared to untreated WT mice, ** is significant change compared to T4-treated and untreated WT mice, **#** is significant change compared to untreated transgenic mice, **+** is significant change compared to T4-treated transgenic mice, and **∧** is significant change compared to T4-supplemented transgenic mice that pre-treated with carvedilol (n = 8/group).

Our current data confirmed our previous finding [12] and showed significant increases in myocardial Rac expression in transgenic (0.63±0.03; p<0.05) compared to wild-type (0.38±0.04) mice at basal conditions. In addition, T4 supplementation resulted in increased myocardial Rac expression in wild-type (0.66±0.04; p<0.01) compared to untreated adult wild-type mice, and resulted in further increase in myocardial Rac expression in transgenic mice (1.20±0.03; p<0.001) compared to all other groups. Pre-treatment of T4-supplemented mice with carvedilol, a mixed α, β-blocker with antioxidant activity [17], or pravastatin, an antihyperlipidemic with Rac-GTPase inhibiting activity [12] did not significantly (1.1±0.03 and 1.3±0.07, respectively) affect T4-induced myocardial Rac expression in ZmRacD mice (Figure 1A).

Interestingly, our current results demonstrate that T4-induced myocardial Rac expression is associated with corresponding increases in both Rac-GTP activity and O_2_·^−^ production in the mouse hearts. Immunostaining of Rac-GTP and DHE staining of O_2_·^−^ showed significant increases in myocardial Rac-GTP activity and O2·^−^ levels in transgenic mice (7.10±0.40 and 2.50±0.10; p<0.001) compared to adult wild-type mice (0.70±0.10 and 0.90±0.10), respectively, at basal conditions. Similarly, T4-stimulated Rac expression in wild-type mice was associated with significant increases in myocardial Rac-GTP activity and O2·^−^ levels (5.61±0.15; p<0.05 and 2.60±0.15; p<0.001), respectively, compared to untreated wild-type mice. Furthermore, T4-stimulated myocardial Rac expression in transgenic mice was associated with highly significant increases in myocardial Rac-GTP activity and O2·^−^ levels (14.10±2.24 and 3.70±0.15; p<0.001), respectively, compared to all other groups (Figure 1B, C, D, E). On the other hand, our data show that pravastatin (6.40±0.60; p<0.001) but not carvedilol (11.40±0.50) significantly reduced T4-induced myocardial Rac-GTP activity to levels that were not significant from untreated transgenic and T4-treated wild-type mice , while still significantly higher compared to untreated wild-type mice (p<0.01) (Figure 1B, C). Conversely, both carvedilol (1.70±0.17; p<0.001) and pravastatin (1.82±0.21; p<0.001) significantly reduced T4-induced myocardial O2·^−^ to levels that were significantly lower from those of untreated transgenic (p<0.01 and 0.05, respectively) and T4-treated wild-type mice (p<0.01), while still significantly higher compared to untreated wild-type mice (p<0.01) (Figure 1D, E).

### T4-Stimulated Myocardial Rac Expression and Activity Led to Physiologic Cardiac Hypertrophy in Wild-Type Mice and Dilated Cardiomyopathy in Transgenic Mice

Heart weight (HW) and body weight (BW) data are shown in (Table 1). Initially, BW did not significantly change among groups. However, T4-treated wild-type mice demonstrated significant increases in both HW (p<0.001) and HW/BW ratios (p<0.001) compared to untreated wild-type and transgenic mice. Similarly, T4-treated transgenic mice showed significant increases in both HW (p<0.001) and HW/BW ratios (p<0.01) compared to untreated wild-type and transgenic mice. Nonetheless, carvedilol and pravastatin did not significantly affect both HW and HW/BW ratios of T4-treated transgenic mice (Table 1).

**Table 1:** Body Weight and Heart Weight Data.

	WT		ZmRacD		ZmRacD + T4	
	Vehicle	T4	Vehicle	T4	Carvedilol	Pravastatin
**BW (g)**	29±0.95	33±0.74	34±1	35±2.5	28±1.4	29±2.01
**HW (mg)**	116±5.22	178±2.7* #	127±5.2	188±5.2* #	163±13.20* #	168±5.8* #
**HW/BW (mg/g)**	3.74±0.33	5.43±0.16* #	4.01±0.08	5.20±0.10* #	4.90±0.26* #	5.30±0.12* #

* is significant change compared to untreated WT mice, and,

# is significant change compared to untreated transgenic mice, (n = 8/group).

Using echocardiography, cardiac dimensions and function were evaluated in wild-type and transgenic mice under basal conditions, as well as after T4 stimulation (Table 2). Our current results show no significant differences in cardiac dimensions and function between adult transgenic and wild-type mice at basal conditions. Likewise, T4-treated wild-type mice demonstrated non-significant changes in cardiac dimensions and function compared to untreated wild-type mice. However, these mice showed significant increases in both LV mass (p<0.001) and LV mass/BW ratio (p<0.05) compared to untreated wild-type and transgenic mice. In contrast, T4-treated transgenic mice experienced remarkable cardiac dysfunction, as shown by decreased LV ejection fraction (EF) (p<0.001) and fractional shortening (FS) (p<0.001) and exhibited enlarged left chambers, as shown by increased systolic (p<0.001) and diastolic (p<0.001) internal diameters, compared to all other groups. Furthermore, these T4-treated mice demonstrated significantly smaller posterior wall thickness during diastole compared to untreated and T4-treated wild-type (p<0.01) and untreated transgenic (p<0.001) mice. Moreover, there were significant increases in both LV mass (p<0.001) and LV mass/BW ratio (p<0.001) compared to untreated wild-type and transgenic mice. Of note, extending the treatment period of T4 to 3 weeks in this current study slightly increased the pathogenic effect of T4 on the transgenic mice; however, this did not reach significance compared to the 2 weeks T4 treatment used in our previous report [12] (Figure S1).

**Table 2:** Ech Table 2. Echocardiography Analysis.

	WT		ZmRacD		ZmRacD + T4	
	Vehicle	T4	Vehicle	T4	Carvedilol	Pravastatin
**% LVEF**	69±3.6	74±3.3	71±2.4	35±1.6** #	65±2.33+	64±6.43+
**% LVFS**	42±2.4	42±2.9	39±1.5	16±1.23** #	34±1.87+	35±4.73+
**IVS,s (mm)**	1.3±0.12	1.4±0.05	1.43±0.04	1.24±0.07	1.5±0.04	1.38±0.09
**IVS,d (mm)**	0.8±0.08	0.98±0.08	1.04±0.04	0.92±0.09	1.01±0.05	1.05±0.09
**LVID,s (mm)**	2.6±.09	3±0.16	2.7±0.11	3.8±0.12** #	2.95±0.27+	2.98±0.19+
**LVID,d (mm)**	3.7±0.11	4.1±0.06	3.94±0.02	4.9±0.13** #	3.96±0.21+	3.96±0.16+
**LVPW,s (mm)**	1.3±0.1	1.5±0.1	1.43±0.09	1.18±0.07	1.43±0.07	1.15±0.09
**LVPW,d (mm)**	1.08±0.1	1.1±0.03	1.12±0.04	0.74±0.04** #	1.05±0.06+	1.00±0.05+
**LV mass (mg)**	100±4.1	143±6.1* #	103±2.8	160±2.03* #	135±7.03* #	142±9.82* #
**LV mass/Body Weight (mg/g)**	2.62±0.12	4.2±0.2* #	2.63±0.12	5.2±0.5* #	4.60±0.34* #	4.72±0.44* #

IVS, interventricular septum; LVID, left ventricular internal diameter; LVPW, left ventricular posterior wall; s, systole; d; diastole; BW, body weight; EF, ejection fraction; FS, fractional shortening.

* is significant change compared to untreated WT mice,

** is significant change compared to T4-treated and untreated WT mice,

# is significant change compared to untreated transgenic mice, and.

+ is significant change compared to T4-treated transgenic mice (n = 8/group).

Echocardiography analysis demonstrated that both carvedilol and pravastatin significantly improved the cardiac functions of the T4-treated transgenic mice as indicated by increased EF (p<0.001) and FS (p<0.001). In addition, carvedilol and pravastatin significantly reduced the LV chamber dilation in T4-treated transgenic mice as evident by decreased systolic (p<0.05) and diastolic (p<0.001) internal diameters. Furthermore, carvedilol and pravastatin significantly increased posterior wall thickness during diastole (p<0.01 and p<0.05), respectively, in T4-treated transgenic mice. On the contrary, neither carvedilol nor pravastatin significantly affected LV mass or LV mass/BW ratios in T4-treated transgenic mice (Table 2).

It is worth mentioning that our recent experiments with propranolol, a β-blocker lacking the antioxidant activity [20], showed inferior efficacy compared to carvedilol. Propranolol was unable to improve the cardiac functions of T4-treated transgenic mice; however, it significantly decreased the LV internal diameter to a remarkably lower extent compared to carvedilol (Figure S2).

### Structural Remodeling of Cardiac Myocytes in the Hearts of T4-Treated Mice

**Figure 2:**
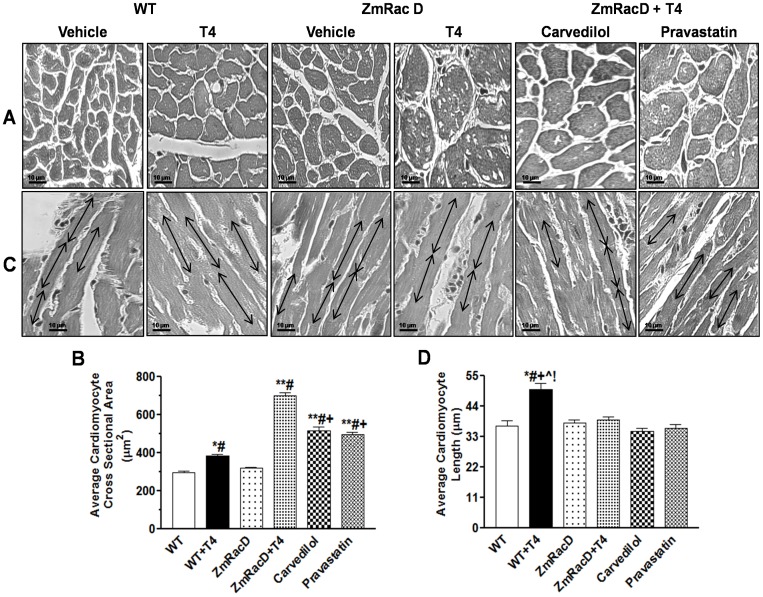
Structural Remodeling of Cardiac Myocytes in the Hearts of T4-Treated Mice. Masson's trichrome staining images with representative bar graphs for average cardiac myocytes cross sectional areas (**A & B**). Masson's trichrome staining images with representative bar graphs for average cardiac myocytes lengths (**C & D**); Magnifications are the same for all panels (size bar, 10 µm). * is significant change compared to untreated WT mice, ** is significant change compared to T4-treated and untreated WT mice, **#** is significant change compared to untreated transgenic mice, **+** is significant change compared to T4-treated transgenic mice, **∧, !** are significant change compared to T4-supplemented transgenic mice that pre-treated with carvedilol and pravastatin, respectively, (n = 8/group).

Both transversal and longitudinal sections through cardiomyocytes stained with Masson's trichrome were obtained and analyzed. For the transversal sections we measured the cross sectional area of the cardiomyocytes. Our results show no statistical difference between wild-type mice (297±9 µm^2^) and transgenic mice (320±5 µm^2^) under basal conditions. However, a statistically significant increase was observed in T4-treated wild-type mice (385±8 µm^2^) compared to untreated wild-type and transgenic mice (p<0.01). In striking contrast to all groups, T4-treated transgenic mice exhibited a dramatically significant increase in the cross sectional area (702±14 µm^2^; p<0.001) (Figure 2A, B). In addition, we noticed that many of the T4-treated transgenic cardiomyocytes appeared vacuolated (Figure 2A). Interestingly, carvedilol (518±20 µm^2^; p<0.001) and pravastatin (498±10 µm^2^; p<0.001) markedly decrease the enlarged cardiomyocytes of T4-treated transgenic mice. However, they did not normalize the cardiomyocyte size and it was still significantly higher (p<0.001) compared to all other groups (Figure 2A, B). Besides, carvedilol and pravastatin clearly decrease the cardiomyocyte vacuolation (Figure 2A).

Cardiomyocyte lengths were measured in those sections with clearly delineated intercalated discs at both ends. We found no significant differences between wild-type (37±2 µm) and transgenic (38±1 µm) mice under basal conditions. In contrast, T4 stimulation resulted in a significant increase in the cardiomyocyte length of wild-type mice (50±3 µm; p<0.001) compared to all other groups. Unexpectedly, T4-treated transgenic mice showed no significant difference in the cardiomyocyte length (38±1 µm) compared to untreated wild-type and transgenic mice. Likewise, carvedilol (35±1 µm) and pravastatin (36±2 µm) did not significantly change the lengths of T4-treated transgenic mice (Figure 2C, D).

### Increased Myocardial Fibrosis in Dilated Cardiomyopathy of T4-Treated Transgenic mice

**Figure 3:**
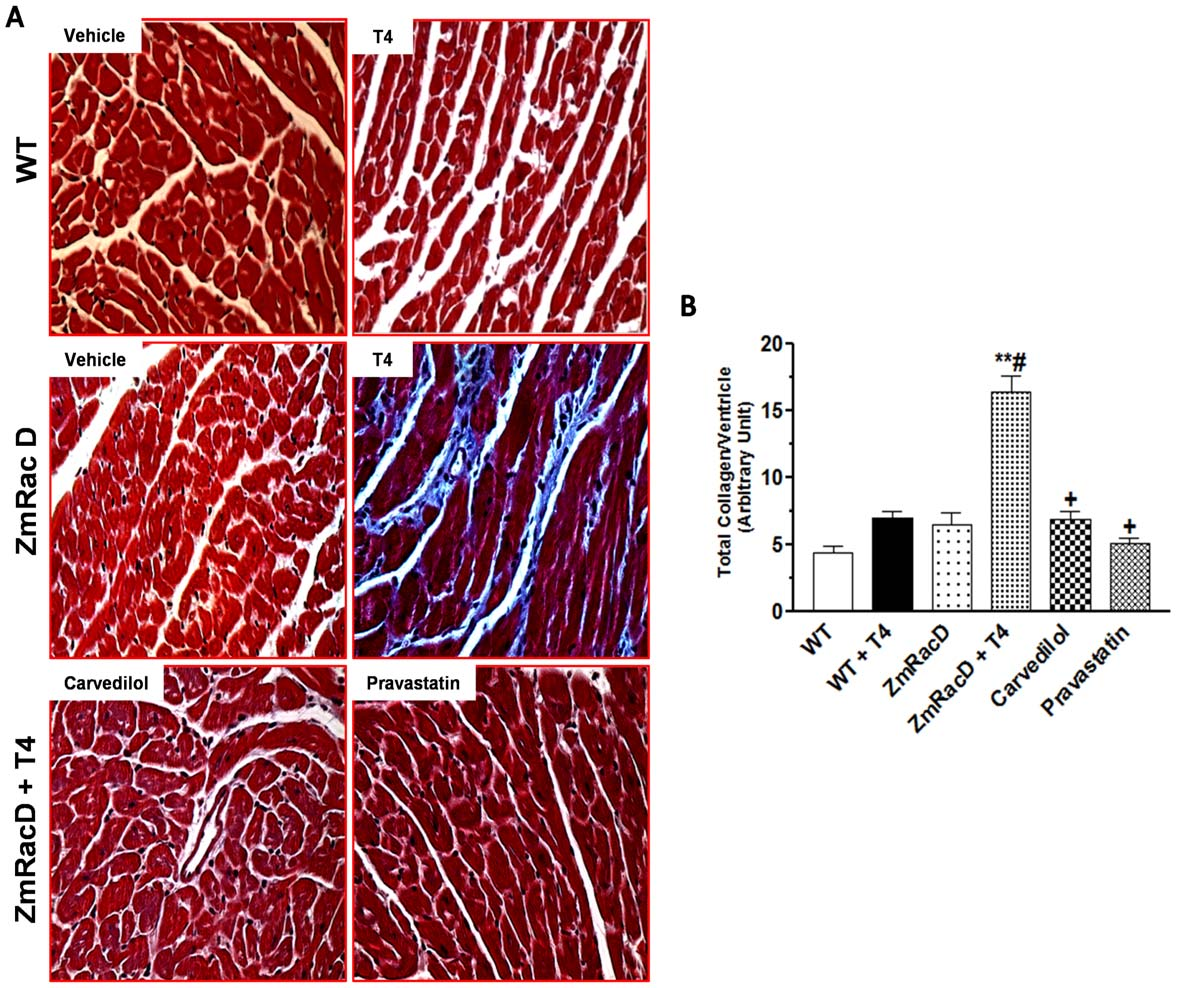
Increased Myocardial Fibrosis in Dilated Cardiomyopathy of T4-Treated Transgenic mice. Masson's trichrome staining shows distinct interstitial fibrosis in the hearts of T4-treated transgenic hearts (**A**); Magnifications are the same for all panels. Representative bar graph showing quantitative results on total collagen deposition in mice hearts (**B**). * is significant change compared to untreated WT mice, ** is significant change compared to T4-treated and untreated WT mice, **#** is significant change compared to untreated transgenic mice, and **+** is significant change compared to T4-treated transgenic mice (n = 8/group).

As evident by Masson's trichrome staining our results showed no apparent fibrosis in wild-type mice under basal conditions (4.40±0.50). Also, the total collagen content in the hearts of untreated transgenic (6.50±0.95) and T4-treated wild-type (7.00±0.53) mice did not significantly changed compared to untreated wild-type mice. Conversely, Masson's trichrome staining clearly demonstrated significantly higher levels of cardiac fibrosis in the ventricles of T4-treated transgenic mice with extremely significant higher total collagen content (16.40±1.20; p<0.001) compared to all other groups (Figure 3A, B). In contrast, carvedilol (6.90±0.60; p<0.001) and pravastatin (5.10±0.40; p<0.001) significantly attenuated the cardiac fibrosis and decreased the total collagen content in the hearts of T4-treated transgenic mice to levels that were not significant from those of all other groups (Figure 3A, B).

### Differential Activation of MAPK in the Cardiac Remodeling of T4-treated Mice

**Figure 4:**
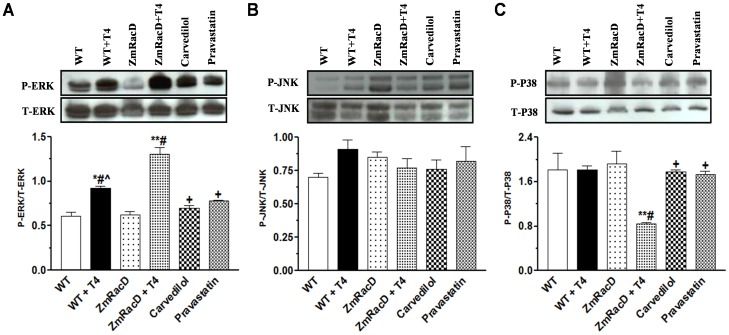
Differential Activation of MAPK in the Cardiac Remodeling of T4-treated Mice. Western blot analyses of total and phospho-ERK1/2 (**A**), total and phospho-JNK (**B**), and total and phospho-p38 (**C**) in the mice hearts. * is significant change compared to untreated WT mice, ** is significant change compared to T4-treated and untreated WT mice, **#** is significant change compared to untreated transgenic mice, **+** is significant change compared to T4-treated transgenic mice, **∧** is significant change compared to T4-supplemented transgenic mice that pre-treated with carvedilol (n = 8/group).

Unregulated Ras/MAPK signaling can result in both cardiac hypertrophy and pathological remodeling in the heart [8]. Therefore, we sought to assess the activation of three key kinases in the MAPK cascade; ERK1/2, JNK, and p38-MAPK in the hearts of both wild-type and transgenic mice under basal conditions, and after T4 stimulation. Our results showed no significant difference in ERK1/2 activation between wild-type (0.60±0.04) and transgenic mice (0.62±0.04) under basal conditions. In contrast, T4 supplementation resulted in a moderate but significant increase in ERK1/2 activation in wild-type mice (0.92±0.03; p<0.01) compared to untreated wild-type and transgenic mice. Interestingly, T4-treated transgenic mice exhibited a highly significant increase in ERK1/2 activation (1.31±0.07; p<0.001) compared to all other groups (Figure 4A). Conversely, carvedilol (0.70±0.03; p<0.001) and pravastatin (0.78±0.01; p<0.001) significantly decreased the activation of ERK in the hearts of T4-treated transgenic mice to levels that were not significantly different from those of untreated wild-type and transgenic mice. Additionally, ERK activity in the hearts of T4-supplemented transgenic mice that pre-treated with carvedilol but not pravastatin showed slight but significant (p<0.05) decrease compared to T4-treated wild-type mice (Figure 4A).

On the other hand, we found no significant difference in the JNK activation in the hearts of both wild-type and transgenic mice under basal conditions (0.71±0.03 and 0.85±0.04), or after T4 stimulation (0.91±0.07 and 0.77±0.07), respectively. Similarly, there were no significant changes in JNK activation in the hearts of T4-supplemented transgenic mice that pre-treated with carvedilol (0.76±0.07) or pravastatin (0.82±0.11) (Figure 4B).

Furthermore, p38-MAPK activation was not significantly different between wild-type (1.80±0.30) and transgenic mice (1.92±0.23) under basal conditions. Similarly, T4-treated wild-type mice showed no significant difference in the activation of this kinase (1.82±0.07) compared to untreated wild-type and transgenic mice. In contrast, T4 treatment resulted in a significant decrease in p38-MAPK activation in the hearts of transgenic mice (0.84±0.03) compared to untreated wild-type (p<0.05), transgenic (p<0.01) and T4-treated wild-type (p<0.01) mice. In contrast, carvedilol (1.78±0.03; p<0.05) and pravastatin (1.73±0.06; p<0.05) significantly increased the activation of p38-MAPK in the hearts of T4-treated transgenic mice to levels that were not significant from those of all other groups (Figure 4C).

### Dilated Cardiomyopathy of T4-Treated Transgenic Mice is Associated with increased Expression of Caspase-8 and Activation of Caspase-3 along with Enhanced Myocardial Apoptosis and TNF-α expression

**Figure 5:**
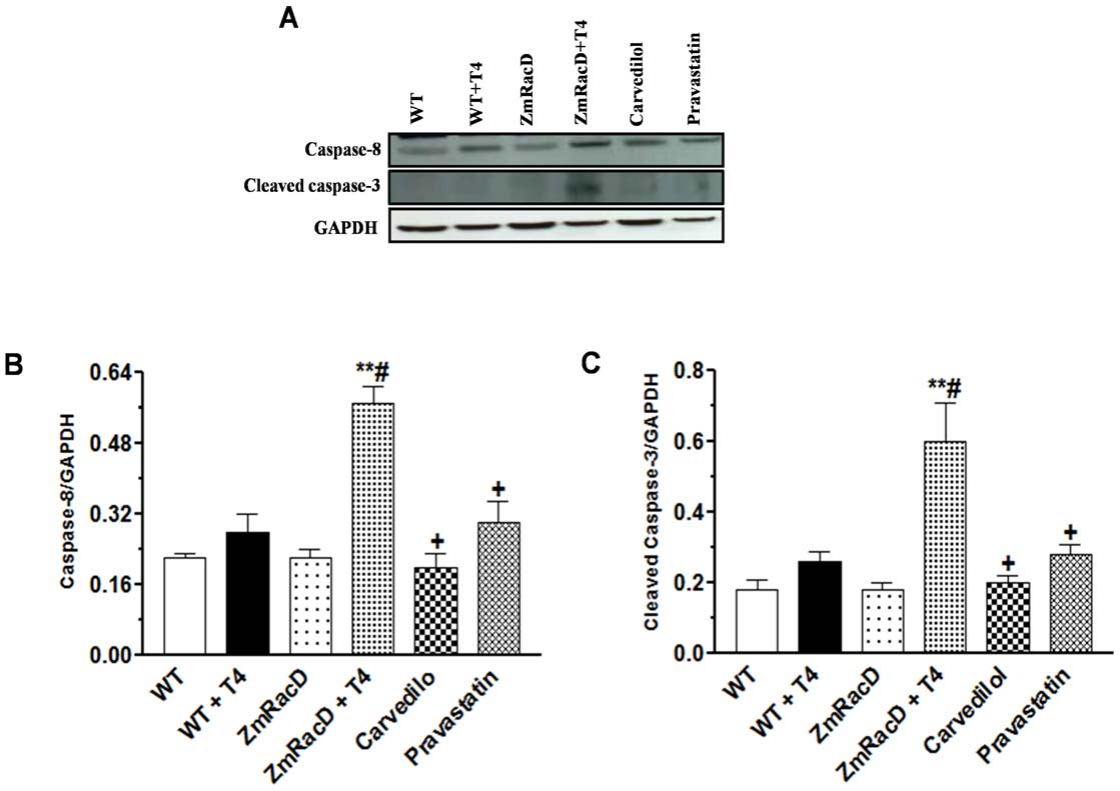
Dilated Cardiomyopathy of T4-Treated Transgenic Mice is Associated with Increased Expression of Caspase-8 and Activation of Caspase-3. Representative images for western blot analysis (**A**) with bar graphs for caspase-8 expression (**B**) and cleaved caspase-3 (**C**) in the mice hearts. * is significant change compared to untreated WT mice, ** is significant change compared to T4-treated and untreated WT mice, **#** is significant change compared to untreated transgenic mice, and **+** is significant change compared to T4-treated transgenic mice (n = 8/group).

Recently, initiator caspase-8 and activating caspase-3 have been reported to play a role in pathological cardiac hypertrophy, cardiac dilation and failure [21], [22]. Therefore, we sought to assess the role of these two key caspases in the cardiac hypertrophy and cardiac dilation developed in wild-type and transgenic mice, respectively, after T4 treatment. Our results showed no significant difference in caspase-8 levels between wild-type (0.20±0.01) and transgenic mice (0.22±0.02) under basal conditions. Similarly, T4-treated wild-type mice showed no significant difference in the expression of this caspase (0.28±0.04) compared to untreated wild-type and transgenic mice. However, T4 treatment resulted in a significant increase in caspase-8 expression in the hearts of the transgenic mice (0.57±0.04; p<0.001) compared to all other groups. In contrast, carvedilol (0.20±0.03; p<0.001) and pravastatin (0.30±0.05; p<0.001) significantly decreased caspase-8 expression in the hearts of T4-treated transgenic mice to levels that were not significant from those of all other groups (Figure 5A, B).

Likewise, there was no significant difference in activated caspase-3 between wild-type (0.20±0.03) and transgenic mice (0.18±0.02) under basal conditions. Also, T4-treated wild-type mice showed no significant difference in the expression of this caspase (0.26±0.03) compared to untreated wild-type and transgenic mice. Nevertheless, T4 treatment resulted in a highly significant increase in cleaved caspase-3 in transgenic mice (0.60±0.11) compared to untreated wild-type (p<0.001), transgenic (p<0.001) and T4-treated wild-type (p<0.01) mice. Conversely, carvedilol (0.20±0.02; p<0.001) and pravastatin (0.28±0.03; p<0.01) significantly decreased caspase-3 activity in the hearts of T4-treated transgenic mice to levels that were not significant from those of all other groups (Figure 5A, C).

**Figure 6:**
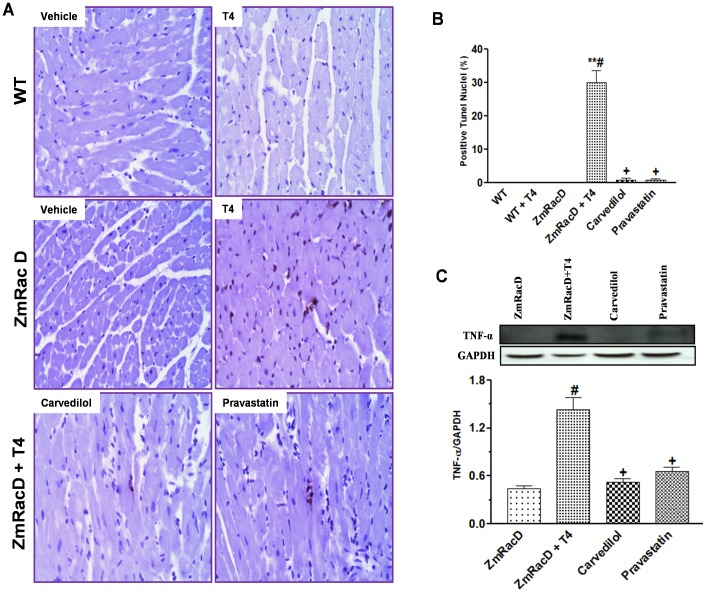
Dilated Cardiomyopathy of T4-Treated Transgenic Mice is Associated with Increased Cardiomyocyte Apoptosis and TNF-α expression. Representative images of in situ terminal deoxynucleotidyl transferase-mediated nick-end labeling (TUNEL) staining in FFPE mid-ventricle sections, with bar graph for the mean % of the brown colored apoptotic nuclei (**A & B**). Western blot analysis of TNF-α in transgenic mice hearts (**C**). * is significant change compared to untreated WT mice, ** is significant change compared to T4-treated and untreated WT mice, **#** is significant change compared to untreated transgenic mice, and **+** is significant change compared to T4-treated transgenic mice (n = 8/group).

To confirm the presence of cardiomyocyte apoptosis we performed TUNEL assays. Our results show that TUNEL-positive nuclei were absent in the hearts of untreated wild-type, untreated transgenic and T4-treated wild-type mice, whereas the number of TUNEL-positive nuclei was markedly increased in the hearts of T4-treated transgenic mice (30±3.60%; p<0.001) compared to all other groups. Then again, carvedilol (0.78±0.68%; p<0.001) and pravastatin (0.77±0.41%; p<0.001) significantly decreased TUNEL-positive nuclei in the hearts of T4-treated transgenic mice to levels that were not significant from that of all other groups (Figure 6A, B).

Since ROS can induce cardiomyocyte apoptosis partly through TNF-α [23], we assessed the level of this cytokine in the hearts of T4-treated and untreated transgenic mice. Our results showed significant increases in TNF-α expression in the hearts of T4-treated transgenic mice (1.43±0.16; p<0.001) compared to untreated transgenic mice (0.40±0.04). In contrast, both carvedilol (0.52±0.05; p<0.01) and pravastatin (0.66±0.05; p<0.01) significantly attenuated this increase to levels that were not significant from that of untreated transgenic mice (Figure 6C).

## Discussion

The pathways inducing the critical transition from compensated hypertrophy to cardiac dilation and failure remain poorly understood. In the present study, we took advantage of two cardiac models; a model of physiologic cardiac hypertrophy and another one of cardiac dilation, that were developed after T4 administration to wild-type and ZmRacD transgenic mice, respectively. This allowed us to investigate the functional, structural and molecular changes associated with these distinct phenotypes. These cardiac models will help us to understand the role of Rac-induced signaling in the development and progression of cardiac remodeling, and to identify the mechanism(s) involved in the transition from cardiac hypertrophy into dilation and failure.

Recently, we have shown the conservation of Rho/Rac proteins in both plant and animal kingdoms *in-vivo* [12]. We showed that over-expression of a constitutively active form of ZmRacD specifically in the hearts of the transgenic mice resulted in cardiac hypertrophy and moderate decrease in systolic function in older mice. Additionally, we showed the increased myocardial Rac expression after T4 treatment in wild-type mice for 2 weeks. Confirming reported data [10], the same T4 treatment of ZmRacD mice up-regulated the α-MHC promoter and led to a higher expression level of ZmRacD transgene [12]. Besides, we illustrated that T4 treatment did not alter the cardiac function and LV internal dimensions in wild-type mice. However, it resulted in cardiac dilation and severe systolic dysfunction in adult transgenic mice within 2 weeks as evident by echocardiography analysis [12]. Notably, in this current report the study duration was extended to 3 weeks instead of 2 weeks [12] based on previous studies demonstrating that the largest part (70–80%) of the myocardial remodeling process in rodent e.g. rat occurs within the first 3 weeks [15]. Although this extended treatment resulted in further decrease in cardiac function and worsened the LV remodeling in the transgenic mice, these changes were not significantly different compared to the 2 weeks treatment used in our previous report [12]. Similar to this finding Kuzman et al. [24] showed that T4 treatment (500 µg/Kg) for 2 weeks resulted in cardiac hypertrophy in wild-type mice, and the same cardiac growth was maintained after extending the treatment period for 4 weeks.

It is becoming progressively clear that Rac, Rac-GTP activity, and Rac-mediated production of O_2_·^−^ play a key role in cardiac remodeling and heart failure [9]–[12]. In agreement with these studies, our current results showed marked increases in Rac expression, Rac-GTP activity and O_2_·^−^ generation in the hearts of T4-treated wild-type mice with higher increases in the hearts of the transgenic mice. Interestingly, these graded increases in Rac-GTP activity and O_2_·^−^ levels were associated with a graded phenotype shift in the heart, from growth and hypertrophy at a low level of Rac-mediated O_2_·^−^ in wild-type mice, to apoptosis and dilation at the higher level of Rac-mediated O_2_·^−^ in transgenic mice. This key finding is in line with previous reports showing that graded rises in the level of myocyte oxidative stress *in-vitro* either directly [25], or indirectly via stretch amplitude [26], provoke a graded shift in cardiac myocyte phenotype, from hypertrophy at low levels of oxidative stress, to apoptosis at higher levels.

In T4-treated wild-type mice, activated Rac/ROS signaling resulted in cardiac hypertrophy as evidenced by increased HW/BW and LV mass/BW ratios. This finding was confirmed by histopathology analysis that showed parallel increases in cardiomyocyte lengths and cross sectional areas. Interstitial fibrosis was absent and cardiac function was normal in these mice signifying a state of typical physiologic cardiac hypertrophy as described by Heineke and Molkentin [1]. In agreement with our finding, previous reports showed the development of physiologic cardiac hypertrophy in rodents after T4 treatment and the involvement of redox signaling in the T4-induced cardiac hypertrophy [27]–[29]. In contrast to our results, Kuzman et al. [29], showed that T4-induced physiologic cardiac hypertrophy in rats was associated with improved cardiac function. This exaggerated response may be due to the higher T4 dose (1 mg/kg) that has been used in this study [29]. However, this latter study as well as our data are in agreement that in hypertrophic cardiomyopathy systolic function is typically normal or enhanced [30].

On the other hand, activated Rac/ROS signaling in the hearts of T4-treated transgenic mice resulted in enlarged LV chambers and severe systolic dysfunction, as evident by increased systolic and diastolic LV internal diameters and decreased LV EF and FS. Furthermore, there was a remarkable decrease in LV posterior wall thickness during diastole. These echocardiogrphic changes are consistent with a typical state of dilated cardiomyopathy [30]. The anatomic origin of cardiac dilation has been proposed to be due to: 1) a slight lengthening of cardiac myocyte, 2) slippage of the cardiac myocytes with subsequent thinning of ventricular wall, and 3) cardiac myocytes loss with subsequent replacement by scar tissue. Additionally, myocardial fibrosis seems to be a main component of dilated cardiomyopathy [31]. Although the increase in myocyte length with increased length/width ratio, are the most commonly observed anatomic changes in dilated cardiomyopathy, other studies showed that cardiac dilation was associated with decreased cardiac myocyte length and unaltered cross sectional area [32] or unchanged cardiac myocyte length and cross sectional area [33]. In the two latter studies [32], [33], the incidence of cardiac dilation was attributed to the slippage of cardiac myocytes with subsequent thinning of ventricular wall. At variance with these studies, cardiac dilation in T4-treated ZmRacD mice was coupled with insignificant change in cardiomyocyte length but highly significant increase in cross sectional area. So, the question remains to be answered: what is the cause of dilated cardiomyopathy in T4-treated ZmRacD mice? While, slippage of cardiac myocytes cannot be excluded, we anticipate that cardiac dilation in T4-treated ZmRacD mice mainly occurred due to cardiac myocyte loss and increased myocardial fibrosis. In support of this hypothesis, our results demonstrated extremely significant increases in the total myocardial collagen content and fibrosis in the T4-treated ZmRacD mice. Furthermore, the level of cleaved caspase-3 was increased in the hearts of these mice, suggesting that caspase-3 is activated. This would then imply that cardiac myocyte apoptosis is enhanced in the hearts of these mice. Moreover, the incidence of oncotic cell death cannot be debarred, since some myocytes in the dilated hearts displayed formation of vacuoles corresponded to severe loss of sarcomere (myolysis) with accumulation of abundant mitochondria and mild increase in infiltration of inflammatory cells [32], [34], [35]. We consistently observed a marked increase in the number of TUNEL-positive cells in the hearts of T4-treated ZmRacD mice. Whether the TUNEL-positive cells in the hearts of T4-treated ZmRacD mice stand for apoptosis or oncosis remains to be revealed. In agreement with our findings, increased apoptosis, oncosis and interstitial fibrosis have been previously reported in transgenic mice and human patients with cardiac dilation [32], [34], [35].

In harmony with the concept that dilated cardiomyopathy is a hypertrophic myopathy in which myocyte growth seems unable to regularize the rise in mural stress formed by the interaction of hemodynamic factors and ventricular anatomical properties [31]; cardiac dilation in T4-treated ZmRacD mice was associated with significant amount of myocyte cellular hypertrophy which resulted in increased cardiac muscle mass as indicated by increased HW/BW and LV mass/BW ratios. Compensatory hypertrophy associated with cardiac dilation was proposed to normalize wall stress, preserve systolic force generation, and compensate for loss of cardiac mass caused by apoptosis [32], [36]. Furthermore, a greater hypertrophic response may partially explain why particular hearts did not exhibit early failure, as proposed by the inspection that the compensated phase of ventricular remodeling leans to be of a longer duration in hearts with greater LV weights [37]. Therefore, we suggest that cardiac hypertrophy associated with the cardiac dilation in the T4-treated ZmRacD mice could explain the survival of these mice. In agreement with our results, cardiac hypertrophy has been reported to be associated with dilated cardiomyopathy in both mice [10] and humans [31].

Another question needs to be answered: what is the mechanism of the profound systolic dysfunction occurred in T4-induced cardiac dilation in ZmRacD mice? Previous reports showed that tissue injury with myocyte cell death and collagen accumulation is followed by a progressive decline of cardiac pump function in aging and in numerous cardiovascular disorders [31]. In addition, it has been reported that TNF-α is a well established mediator to impair cardiac function and it is implicated in different cardiac pathologies [38]–[40]. TNF-α can impair heart function either directly through its negative inotropic effect [38] or indirectly through inducing apoptosis [39] or activating cardiac caspases e.g. caspase-8 and caspase-3 without significant cell death [40]. Activation of caspases may initiate the progressive cleavage of myocardial contractile proteins, or it may activate other proteolytic enzymes e.g. matrix metalloproteinase that can degrade myocardial extracellular protein with subsequent decline in LV systolic function [40]. In line with these studies our current results presented that increased TNF-α, cardiac caspases, myocyte apoptosis and myocardial fibrosis are key players in the development of LV systolic dysfunction in T4-induced cardiac dilation in ZmRacD mice.

Among the most conserved signal transduction systems in the heart is the MAPK cascade. It involves consecutively acting protein kinases leading to the activation of three terminal MAPKs; ERK, JNK, and p38 kinase. Once activated, these kinases phosphorylate a wide range of intracellular targets that include various transcription factors leading to reprogramming of gene expression. Unregulated MAPK signaling can result in both cardiac hypertrophy and pathological remodeling in the heart [8]. Here we show that T4-induced cardiac hypertrophy in wild-type mice was coupled with increased activation of ERK1/2; however, there was no significant change in the activities of both JNK and p38-MAPK. Similarly, Kuzman et al. [29], showed that T4-induced cardiac hypertrophy in rats was associated with unchanged JNK and p38-MAPK activities. Yet, these rats also exhibited no significant change in the ERK1/2 activity either [29], while Araujo et al. [41], showed that T4-induced cardiac hypertrophy in rats was associated with increased ERK1/2 but not increased JNK activity. Low levels of oxidative stress were also found to be involved in cardiac myocyte hypertrophy, with increased ERK1/2 but not JNK activity *in-vitro* [26].

To date, reported results on MAPKs activity in failing myocardium are inconsistent. Various reports showed either increased ERK1/2, JNKs and p38-MAPK activities, increased JNK and p38-MAPK activities, but not ERK1/2 activity, or decreased p38-MAPK activity [42]. Our current data showed that T4-induced dilated cardiomyopathy in ZmRacD mice was associated with increased ERK1/2 activity and down-regulation of p38-MAPK activity; however, there was no significant change in JNK activity. Compatible with our findings increased ERK1/2 and decreased p38-MAPK activities have been previously reported in failing myocardium both in animals [43], [44] and humans [42], [45], [46]. JNK activity has been shown to be either up-regulated [43] or down-regulated [42], [44] in failing myocardium, while our results showed unchanged JNK activity in dilated hearts of ZmRacD mice.

It is worth noting that ERK1/2 activity is significantly higher, however, p38-MAPK activity is significantly lower in T4-induced cardiac dilation in ZmRacD mice compared to their corresponding activities in T4-induced cardiac hypertrophy in wild-type mice. This may indicate an important role for both kinases in the transition from physiologic cardiac hypertrophy to cardiac dilation and failure. Yet, this remains to be determined.

Apoptosis has been coupled with numerous forms of heart failure, e.g. myocarditis, ischemia/reperfusion injury, and congestive heart failure. Two major apoptotic pathways have been reported, and both congregate on the activation of caspases [40]. Caspase-8 is one of the most upstream caspases involved in cardiac myocyte death. Transgenic mice that express a conditionally cardiac-specific active caspase-8 exhibited a lethal dilated cardiomyopathy [22]. In addition, caspase-3 is a downstream effecter of caspase-8 in apoptotic cell death [47]. Increased caspase-3 was evident in pathological LV hypertrophy with LV dysfunction and LV dilation, but not in physiological hypertrophy [21], [32], [36]. Additionally, increased Rac/ROS activity resulted in LV remodeling and increased caspase-3 expression [48]. Consistent with these findings we show here increased caspase-8 expression and caspase-3 activity in T4-induced cardiac dilation in ZmRacD mice, whereas there were no significant differences in both caspases in T4-induced physiologic cardiac hypertrophy in wild-type mice.

It has been demonstrated that ROS can induce cardiomyocyte apoptosis partially through TNF-α [23]. The TNF-α mediated pathway has been shown to be responsible for myocardial caspase-3 and -8 activation [40]. On the other hand, it has been reported that p38-MAPK may protect cardiac myocyte from apoptosis and p38 knockout mice exhibit increased apoptosis and caspase-3 activity compared to control [33]. Furthermore, it has been recently shown that activation of ERK1/2 contributes to cell death in some cell types and organs and it may act upstream to TNF-α, caspase-8 or caspase-3 in the apoptotic pathway [47]. In the present study one or more of these effectors (i.e. increased TNF-α and ERK1/2, or decreased p38-MAPK) may be involved in the ROS-induced apoptosis in our model. However, the exact mechanism remains to be elucidated.

To confirm the role of Rac and Rac-mediated O_2_·^−^ generation in the developed cardiac phenotypes in our model, T4-treated transgenic mice were pre-treated with either carvedilol or pravastatin. Carvedilol is a non-selective vasodilating β-blocker working on β_1_-, β_2_-, and α_1_-adrenoceptors. Additionally, Carvedilol and its metabolites are potent antioxidants [17], [20] due to their abilities to 1) scavenge O2·^−^, 2) inhibit O2·^−^ production, 3) attenuate lipid peroxidation, and 4) spare the consumption of endogenous antioxidants [20], [49]. Recently, inhibition of O2·^−^ production by carvedilol has been also accredited to its ability to decrease NADPH oxidase subunits (P^47phox^ and P^67phox^) in the heart [50]. On the other hand, pravastatin is an antihyperlipidemic agent that inhibits Rac-GTPase and NADPH oxidase activities both in animal [12], [51] and human [11] hearts, decreases myocardial O_2_·^−^ levels [52] and stimulates the endogenous antioxidant mechanism in the heart [51]. In our experiments, carvedilol significantly decreased O_2_·^−^ but not Rac-GTPase activity in dilated transgenic mice hearts, while; pravastatin inhibited both. Both treatments attenuated all downstream signaling: ERK1/2 activity, TNF-α expression, caspase-8 expression and caspase-3 activity. Conversely, they increased p38-MAPK activity. This led to marked decrease in cardiac myocyte size and ablation of myocardial fibrosis and apoptosis, with subsequent normalization of systolic and diastolic LV dimensions and preservation of heart functions. Thus, we confirm previous studies that showed the effectiveness of carvedilol [17], [49], [53]–[55] and pravastatin [11], [51], [52], [56] in decreasing oxidative response, TNF-α expression, fibrosis, apoptosis and attenuating cardiac dilation and failure both in animals and humans.

β-blockers are useful antioxidative therapy in patients with heart failure. They exert their antioxidatant effects either via blocking β-adrenoreceptors with subsequent inhibition of catecholamine-induced oxidative stress or via direct antioxidant properties of some β-blockers such as carvedilol [57]. To confirm that the antioxidant activity but not the β-blocking activity of carvedilol is the main contributor in these improvements observed in our model, propranolol, a β-blocker lacking the antioxidant activity, at higher dose (10 mg/kg) failed to improve the LV function of the dilated transgenic hearts, and it only resulted in significantly less improvements in LV dimensions of these hearts compared to carvedilol at the low dose (2 mg/kg) used in this current study. This is consistent with previous studies [20].

Another important effect of carvedilol [17], [54], [55] and pravastatin [56], [58] is the decrease in cardiomyocyte size, HW and LV mass. In contrast, in a rat model of cardiac dilation with compensatory hypertrophy, pravastatin significantly decreased Rac and NADPH oxidase activity leading to inhibition of cardiac dilation, but not LV mass or cardiomyocyte size [51]. Consistent with this latter study [51], our current results showed that carvedilol and pravastatin were unable to significantly decrease HW or LV mass; however both drugs significantly decreased the cardiomyocyte size. This clearly indicates the involvement of Rac-mediated O_2_·^−^ in the development of cardiac hypertrophy in our model. The inability of carvedilol and pravastatin to normalize the HW and LV mass could indicate the involvement of other signaling pathways besides Rac signaling in the development of T4-induced cardiac hypertrophy in these mice such as PI3K, PKB/Akt, or the rennin-angiotensin system [59].

Taken together, our data demonstrate that T4-induced ZmRacD is a novel mouse model of dilated cardiomyopathy that shares many characteristics with the human disease phenotype. To our knowledge, this is the first study to show graded Rac-mediated O_2_·^−^ results in cardiac phenotype shift *in-vivo*. Moreover, Rac-mediated O_2_·^−^ generation, cardiomyocyte apoptosis and myocardial fibrosis seem to play a pivotal role in the transition from cardiac hypertrophy to cardiac dilation and failure. Targeting Rac signaling could represent valuable therapeutic strategy not only in saving the failing myocardium but also to prevent this transition process.

A limitation of the present study is that cardiomyocyte length is generally in the range of 78–127 µm as revealed from isolated cardiomyocytes [60]. However, in this current study cardiomyocyte lengths were obtained from stained cardiac tissue. Smaller cardiomyocyte lengths reported here (35–50 µm), may be partially due to the effect of tissue processing and sectioning. Even though, our data are consistent with other studies [61], that reported the same cardiac cell length (about 35 µm) in wild-type mice of approximate age (5–7 months) to our mice (6–8 months).

## Supporting Information

Figure S1 **Effect of Thyroxine (T4) treatment on the hearts of ZmRacD Mice.** M-mode echocardiography images of the left ventricle (LV) of ZmRacD mice at basal condition (**A**), and after T4-treatment for 1 week (**B**), 2 weeks (**C**) and 3 weeks (**D**). Representative bar graphs for LV ejection fraction (EF) (**E**), fractional shortening (FS) (**F**), internal diameter during systole (LVID, s) (**G**), and internal diameter during diastole (LVID, d) (**H**). * is significant change compared to basal conditions and # is significant change compared to 1 week T4 treatment.
(TIF)

Figure S2 **Comparative Effects of Carvedilol and Propranolol on the Left Ventricle (LV) Function and Internal Diameters of T4-treated ZmRacD Mice.** Representative bar graphs for LV ejection fraction (EF) (**A**), fractional shortening (FS) (**B**), internal diameter during systole (LVID, s) (**C**), and internal diameter during diastole (LVID, d) (**D**). # is significant change compared to untreated ZmRacD mice,+ is significant change compared to T4-treated ZmRacD mice and ∧ is significant change compared to T4-supplemented transgenic mice that pre-treated with carvedilol.
(TIF)

## References

[1] Heineke J, Molkentin JD (2006) Regulation of cardiac hypertrophy by intracellular signalling pathways. Nat Rev Mol Cell Biol 7: 589–600. 16936699 10.1038/nrm1983

[2] Klein L, O'Connor CM, Gattis WA, Zampino M, de Luca L, et al (2003) Pharmacologic therapy for patients with chronic heart failure and reduced systolic function: review of trials and practical considerations. Am J Cardiol 91: 18F–40F. 10.1016/s0002-9149(02)03336-2 12729848

[3] Lips DJ, deWindt LJ, van Kraaij DJ, Doevendans PA (2003) Molecular determinants of myocardial hypertrophy and failure: alternative pathways for beneficial and maladaptive hypertrophy. Eur Heart J 24: 883–896. 12714020 10.1016/s0195-668x(02)00829-1

[4] Berenji K, Drazner MH, Rothermel BA, Hill JA (2005) Does load-induced ventricular hypertrophy progress to systolic heart failure? Am J Physiol Heart Circ Physiol 289: H8–H16. 15961379 10.1152/ajpheart.01303.2004

[5] Diwan A, Dorn GWII (2007) Decompensation of cardiac hypertrophy: cellular mechanisms and novel therapeutic targets. Physiology 22: 56–64. 17289931 10.1152/physiol.00033.2006

[6] Selvetella G, Hirsch E, Notte A, Tarone G, Lembo G (2004) Adaptive and maladaptive hypertrophic pathways: points of convergence and divergence. Cardiovasc Res 63: 373–380. 15276462 10.1016/j.cardiores.2004.04.031

[7] Dorn GW (2009) Novel pharmacotherapies to abrogate postinfarction ventricular remodeling. Nat Rev Cardiol 6: 283–291. 19352332 10.1038/nrcardio.2009.12

[8] Rose BA, Force T, Wang Y (2010) Mitogen-activated protein kinase signaling in the heart: angels versus demons in a heart-breaking tale. Physiol Rev 90: 1507–1546. 20959622 10.1152/physrev.00054.2009 PMC3808831

[9] Lezoualc'h F, Metrich M, Hmitou I, Duquesnes N, Morel E (2008) Small GTP-binding proteins and their regulators in cardiac hypertrophy. J Mol Cell Cardiol 44: 623–632. 18339399 10.1016/j.yjmcc.2008.01.011

[10] Sussman MA, Welch S, Walker A, Klevitsky R, Hewett TE, et al (2000) Altered focal adhesion regulation correlates with cardiomyopathy in mice expressing constitutively active rac1. J Clin Invest 105: 875–886. 10749567 10.1172/JCI8497 PMC377478

[11] Maack C, Kartes T, Kilter H, Schäfers HJ, Nickenig G, et al (2003) Oxygen free radical release in human failing myocardium is associated with increased activity of rac1-GTPase and represents a target for statin treatment. Circulation 108: 1567–1574. 12963641 10.1161/01.CIR.0000091084.46500.BB

[12] Elnakish MT, Awad MM, Hassona MD, Alhaj MA, Kulkarni A, et al (2011) Cardiac Remodeling Caused by Transgenic Over-expression of a Corn Rac Gene. Am J Physiol Heart Circ Physiol 301: H868–H880. 21622832 10.1152/ajpheart.00807.2010

[13] Frey N, Olson EN (2003) Cardiac hypertrophy: the good, the bad, and the ugly. Annu Rev Physiol 65: 45–79. 12524460 10.1146/annurev.physiol.65.092101.142243

[14] Pierres C, Gaugain-Hamidi A (2009) Concentrated Liquid Thyroid Hormone Composition. Patentdocs website. Available: http://www.faqs.org/patents/app/20090270507#ixzz1R067AyTq. Accessed: 2012 August 3.

[15] Pfeffer MA, Braunwald E (1990) Ventricular remodeling after myocardial infarction. Circulation 81: 1161–1172. 2138525 10.1161/01.cir.81.4.1161

[16] Schaefer WH, Politowski J, Hwang B, Dixon F Jr, Goalwin A, et al (1998) Metabolism of carvedilol in dogs, rats, and mice. Drug Metab Dispos 26: 958–969. 9763400

[17] Watanabe K, Ohta Y, Nakazawa M, Higuchi H, Hasegawa G, et al (2000) Low dose carvedilol inhibits progression of heart failure in rats with dilated cardiomyopathy. Br J Pharmacol 130: 1489–1495. 10928949 10.1038/sj.bjp.0703450 PMC1572210

[18] Samuel MS, Lourenço FC, Olson MF (2011) K-Ras mediated murine epidermal tumorigenesis is dependent upon and associated with elevated Rac1 activity. PLoS One 6: e17143. 21358804 10.1371/journal.pone.0017143 PMC3039675

[19] Hassona MD, Elnakish MT, Abouelnaga ZA, Alhaj M, Wani AA, et al (2011) The Effect of Selective Antihypertensive Drugs on the Vascular Remodeling-associated Hypertension: Insights from a Profilin1 Transgenic Mouse Model. J Cardiovasc Pharmacol 57: 550–558. 21326111 10.1097/FJC.0b013e318212b1c2

[20] Feuerstein GZ, Ruffolo RR Jr (1996) Carvedilol, a novel vasodilating beta-blocker with the potential for cardiovascular organ protection. Eur Heart J 17: 24–29. 8733068 10.1093/eurheartj/17.suppl_b.24

[21] Balakumar P, Singh M (2006) The possible role of caspase-3 in pathological and physiological cardiac hypertrophy in rats. Basic Clin Pharmacol Toxicol 99: 418–424. 17169122 10.1111/j.1742-7843.2006.pto_569.x

[22] Wencker D, Chandra M, Nguyen K, Miao W, Garantziotis S, et al (2003) A mechanistic role for cardiac myocyte apoptosis in heart failure. J Clin Invest 111: 1497–1504. 12750399 10.1172/JCI17664 PMC155051

[23] Aikawa R, Nitta-Komatsubara Y, Kudoh S, Takano H, Nagai T, et al (2002) Reactive oxygen species induce cardiomyocyte apoptosis partly through TNF-alpha. Cytokine 18: 179–183. 12126639 10.1006/cyto.2001.1007

[24] Kuzman JA, O'Connell TD, Gerdes AM (2007) Rapamycin prevents thyroid hormone-induced cardiac hypertrophy. Endocrinology 148: 3477–3484. 17395699 10.1210/en.2007-0099

[25] Siwik DA, Tzortzis JD, Pimental DR, Chang DL, Pagano PJ, et al (1999) Inhibition of copper-zinc superoxide dismutase induces cell growth, hypertrophic phenotype, and apoptosis in neonatal rat cardiac myocytes in vitro. Circ Res 85: 147–153. 10417396 10.1161/01.res.85.2.147

[26] Pimentel DR, Amin JK, Xiao L, Miller T, Viereck J, et al (2001) Reactive oxygen species mediate amplitude-dependent hypertrophic and apoptotic responses to mechanical stretch in cardiac myocytes. Circ Res 89: 453–460. 11532907 10.1161/hh1701.096615

[27] Araujo AS, Schenkel P, Enzveiler AT, Fernandes TR, Partata WA, et al (2008) The role of redox signaling in cardiac hypertrophy induced by experimental hyperthyroidism. J Mol Endocrinol 41: 423–430. 18787053 10.1677/JME-08-0024

[28] Florini JR, Saito Y, Manowitz EJ (1973) Effect of age on thyroxin-induced cardiac hypertrophy in mice. J Gerontol 28: 293–297. 4267853 10.1093/geronj/28.3.293

[29] Kuzman JA, Vogelsang KA, Thomas TA, Gerdes AM (2005) L-Thyroxine activates Akt signaling in the heart. J Mol Cell Cardiol 39: 251–258. 15890358 10.1016/j.yjmcc.2005.03.020

[30] Freeman K, Colon-Rivera C, Olsson MC, Moore RL, Weinberger HD, et al (2001) Progression from hypertrophic to dilated cardiomyopathy in mice that express a mutant myosin transgene. Am J Physiol Heart Circ Physiol 280: 151–159. 10.1152/ajpheart.2001.280.1.H151 11123229

[31] Beltrami CA, Finato N, Rocco M, Feruglio GA, Puricelli C, et al (1995) The cellular basis of dilated cardiomyopathy in humans. J Mol Cell Cardiol 27: 291–305. 7760353 10.1016/s0022-2828(08)80028-4

[32] Yamamoto S, Yang G, Zablocki D, Liu J, Hong C, et al (2003) Activation of Mst1 causes dilated cardiomyopathy by stimulating apoptosis without compensatory ventricular myocyte hypertrophy. J Clin Invest 111: 1463–1474. 12750396 10.1172/JCI17459 PMC155047

[33] Nishida K, Yamaguchi O, Hirotani S, Hikoso S, Higuchi Y (2004) P38 alpha mitogen-activated protein kinase plays a critical role in cardiomyocyte survival but not in cardiac hypertrophic growth in response to pressure overload. Mol Cell Biol 24: 10611–10620. 15572667 10.1128/MCB.24.24.10611-10620.2004 PMC533965

[34] Fentzke RC, Korcarz CE, Lang RM, Lin H, Leiden JM (1998) Dilated cardiomyopathy in transgenic mice expressing a dominant-negative CREB transcription factor in the heart. J Clin Invest 101: 2415–2426. 9616213 10.1172/JCI2950 PMC508831

[35] Corradi D, Tchana B, Miller D, Manotti L, Maestri R, et al (2009) Dilated form of endocardial fibroelastosis as a result of deficiency in respiratory-chain complexes I and IV. Circulation 120: e38–40. 19667241 10.1161/CIRCULATIONAHA.108.840660

[36] Philipp S, Pagel I, Höhnel K, Lutz J, Buttgereit J, et al (2004) Regulation of caspase-3 and Fas in pressure overload-induced left ventricular dysfunction. Eur J Heart Fail 6: 845–851. 15556045 10.1016/j.ejheart.2004.01.014

[37] Brower GL, Janicki JS (2001) Contribution of ventricular remodeling to pathogenesis of heart failure in rats. Am J Physiol Heart Circ Physiol 280: H674–H683. 11158966 10.1152/ajpheart.2001.280.2.H674

[38] Yokoyama T, Vaca L, Rossen RD, Durante W, Hazarika P, et al (1993) Cellular basis for the negative inotropic effects of tumor necrosis factor-alpha in the adult mammalian heart. J Clin Invest 92: 2303–2312. 8227345 10.1172/JCI116834 PMC288411

[39] Lu X, Hamilton JA, Shen J, Pang T, Jones DL, et al (2006) Role of tumor necrosis factor-alpha in myocardial dysfunction and apoptosis during hindlimb ischemia and reperfusion. Crit Care Med 34: 484–491. 16424732 10.1097/01.ccm.0000199079.64231.c1

[40] Carlson DL, Willis MS, White DJ, Horton JW, Giroir BP (2005) Tumor necrosis factor-alpha-induced caspase activation mediates endotoxin-related cardiac dysfunction. Crit Care Med 33: 1021–1028. 15891331 10.1097/01.ccm.0000163398.79679.66

[41] Araujo AS, Fernandes T, Ribeiro MF, Khaper N, Belló-Klein A (2010) Redox regulation of myocardial ERK 1/2 phosphorylation in experimental hyperthyroidism: role of thioredoxin-peroxiredoxin system. J Cardiovasc Pharmacol 56: 513–517. 20729758 10.1097/FJC.0b013e3181f50a70

[42] Communal C, Colucci WS, Remondino A, Sawyer DB, Port JD, et al (2002) Reciprocal modulation of mitogen-activated protein kinases and mitogen-activated protein kinase phosphatase 1 and 2 in failing human myocardium. J Card Fail 8: 86–92. 12016632 10.1054/jcaf.2002.32755

[43] Muchir A, Pavlidis P, Decostre V, Herron AJ, Arimura T, et al (2007) Activation of MAPK pathways links LMNA mutations to cardiomyopathy in Emery-Dreifuss muscular dystrophy. J Clin Invest 117: 1282–1293. 17446932 10.1172/JCI29042 PMC1849984

[44] Tenhunen O, Soini Y, Ilves M, Rysä J, Tuukkanen J, et al (2006) p38 Kinase rescues failing myocardium after myocardial infarction: evidence for angiogenic and anti-apoptotic mechanisms. FASEB J 20: E1276–E1286. 10.1096/fj.05-5618fje 16849392

[45] Dong Y, Gao D, Chen L, Lin R, Conte JV, et al (2006) Increased ERK activation and decreased MKP-1 expression in human myocardium with congestive heart failure. J Cardiothorac Ren Res 1: 123–130.

[46] Lemke LE, Bloem LJ, Fouts R, Esterman M, Sandusky G, et al (2001) Decreased p38 MAPK activity in end-stage failing human myocardium: p38 MAPK alpha is the predominant isoform expressed in human heart. J Mol Cell Cardiol 33: 1527–1540. 11448140 10.1006/jmcc.2001.1415

[47] Zhuang S, Schnellmann RG (2006) A death-promoting role for extracellular signal-regulated kinase. J Pharmacol Exp Ther 319: 991–997. 16801453 10.1124/jpet.106.107367

[48] Worou ME, Belmokhtar K, Bonnet P, Vourc'h P, Machet MC, et al (2011) Hemin decreases cardiac oxidative stress and fibrosis in a rat model of systemic hypertension via PI3K/Akt signalling. Cardiovasc Res 91: 320–329. 21406596 10.1093/cvr/cvr072

[49] Yaoita H, Sakabe A, Maehara K, Maruyama Y (2002) Different effects of carvedilol, metoprolol, and propranolol on left ventricular remodeling after coronary stenosis or after permanent coronary occlusion in rats. Circulation 105: 975–980. 11864928 10.1161/hc0802.104503

[50] Arozal W, Watanabe K, Veeraveedu PT, Ma M, Thandavarayan RA, et al (2010) Protective effect of carvedilol on daunorubicin-induced cardiotoxicity and nephrotoxicity in rats. Toxicology 274: 18–26. 20452391 10.1016/j.tox.2010.05.003

[51] Ichihara S, Noda A, Nagata K, Obata K, Xu J, et al (2006) Pravastatin increases survival and suppresses an increase in myocardial matrix metalloproteinase activity in a rat model of heart failure. Cardiovasc Res 69: 726–735. 16165109 10.1016/j.cardiores.2005.08.001

[52] Cheng CF, Juan SH, Chen JJ, Chao YC, Chen HH, et al (2008) Pravastatin attenuates carboplatin-induced cardiotoxicity via inhibition of oxidative stress associated apoptosis. Apoptosis 13: 883–894. 18483861 10.1007/s10495-008-0214-9

[53] Chua S, Sheu JJ, Chang LT, Lee FY, Wu CJ, et al (2008) Comparison of losartan and carvedilol on attenuating inflammatory and oxidative response and preserving energytranscription factors and left ventricular function in dilated cardiomyopathy rats. Int. Heart J 49: 605–619. 10.1536/ihj.49.605 18971572

[54] Li B, Liao YH, Cheng X, Ge H, Guo H, et al (2006) Effects of carvedilol on cardiac cytokines expression and remodeling in rat with acute myocardial infarction. Int J Cardiol 111: 247–255. 16310260 10.1016/j.ijcard.2005.08.065

[55] Palazzuoli A, Bruni F, Puccetti L, Pastorelli M, Angori P, et al (2002) Effects of carvedilol on left ventricular remodeling and systolic function in elderly patients with heart failure. Eur J Heart Fail 4: 765–770. 12453548 10.1016/s1388-9842(02)00114-9

[56] Zhao H, Liao Y, Minamino T, Asano Y, Asakura M, et al (2008) Inhibition of cardiac remodeling by pravastatin is associated with amelioration of endoplasmic reticulum stress. Hypertens Res 31: 1977–1987. 19015605 10.1291/hypres.31.1977

[57] Nakamura K, Murakami M, Miura D, Yunoki K, Enko K, et al (2011) Beta-Blockers and Oxidative Stress in Patients with Heart Failure. Pharmaceuticals 4: 1088–1100. 26791643 10.3390/ph4081088 PMC4058661

[58] Xu Z, Okamoto H, Akino M, Onozuka H, Matsui Y, et al (2008) Pravastatin attenuates left ventricular remodeling and diastolic dysfunction in angiotensin II-induced hypertensive mice. J Cardiovasc Pharmacol 51: 62–70. 18209570 10.1097/FJC.0b013e31815bb629

[59] Ojamaa K (2010) Signaling mechanisms in thyroid hormone-induced cardiac hypertrophy. Vascul Pharmacol 52: 113–119. 20005976 10.1016/j.vph.2009.11.008 PMC2830872

[60] Korecky B, Rakusan K (1978) Normal and hypertrophic growth of the rat heart: changes in cell dimensions and number. Am J Physiol 234: H123–H128. 146437 10.1152/ajpheart.1978.234.2.H123

[61] Helms SA, Azhar G, Zuo C, Theus SA, Bartke A, et al (2010) Smaller cardiac cell size and reduced extra-cellular collagen might be beneficial for hearts of Ames dwarf mice. Int J Biol Sci 6: 475–490. 20827400 10.7150/ijbs.6.475 PMC2935670

