# Supplementary figures and images for: Rac-Induced Left Ventricular Dilation in Thyroxin-Treated ZmRacD Transgenic Mice: Role of Cardiomyocyte Apoptosis and Myocardial Fibrosis
Source: PLoS One. 2012 Aug 24;7(8):e42500. doi: 10.1371/journal.pone.0042500 (PMC3427332; doi:10.1371/journal.pone.0042500)

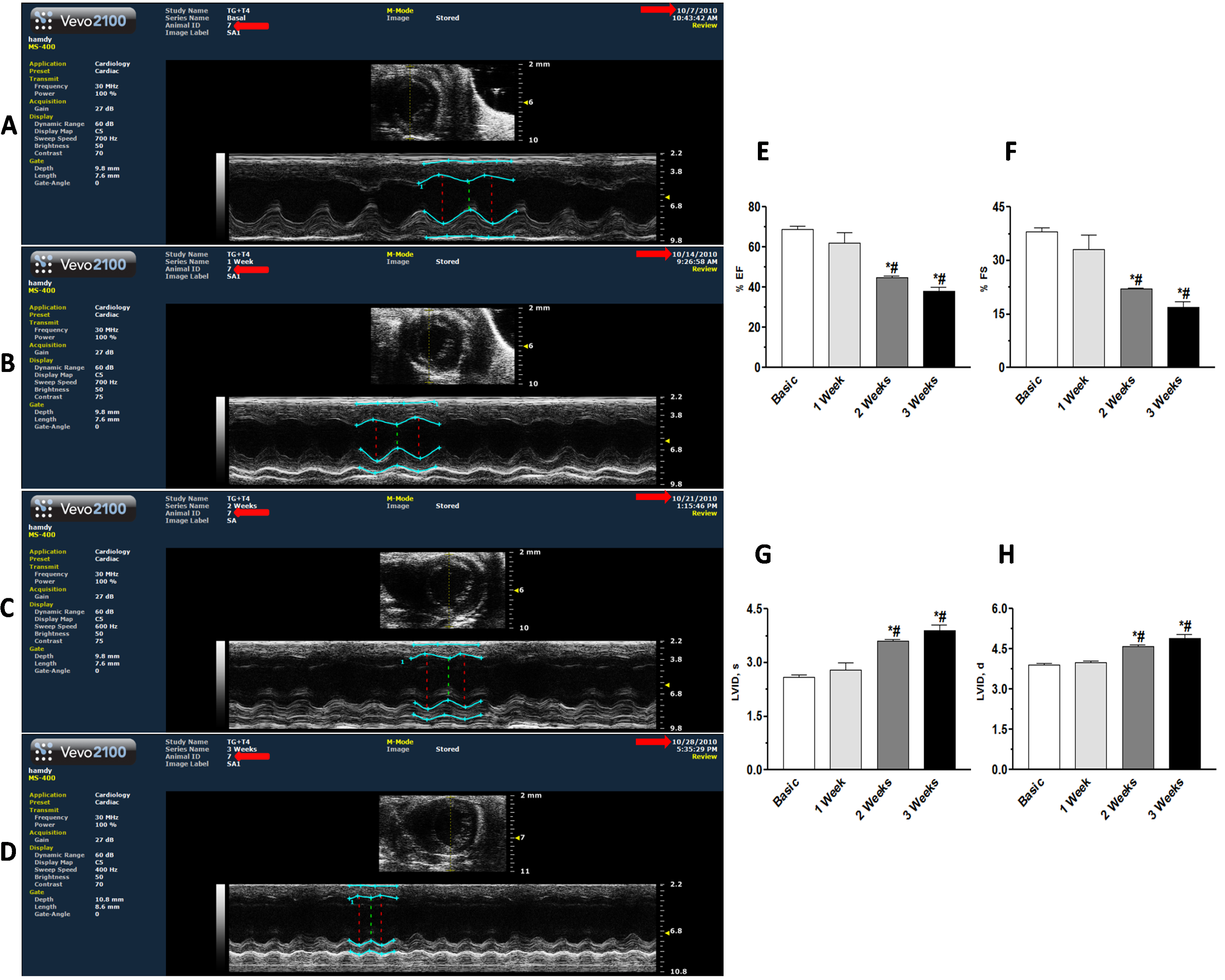

Supplement: Figure S1 — Effect of Thyroxine (T4) treatment on the hearts of ZmRacD Mice. M-mode echocardiography images of the left ventricle (LV) of ZmRacD mice at basal condition (A), and after T4-treatment for 1 week (B), 2 weeks (C) and 3 weeks (D). Representative bar graphs for LV ejection fraction (EF) (E), fractional shortening (FS) (F), internal diameter during systole (LVID, s) (G), and internal diameter during diastole (LVID, d) (H). * is significant change compared to basal conditions and # is significant change compared to 1 week T4 treatment. (TIF) [file pone.0042500.s001.tif]

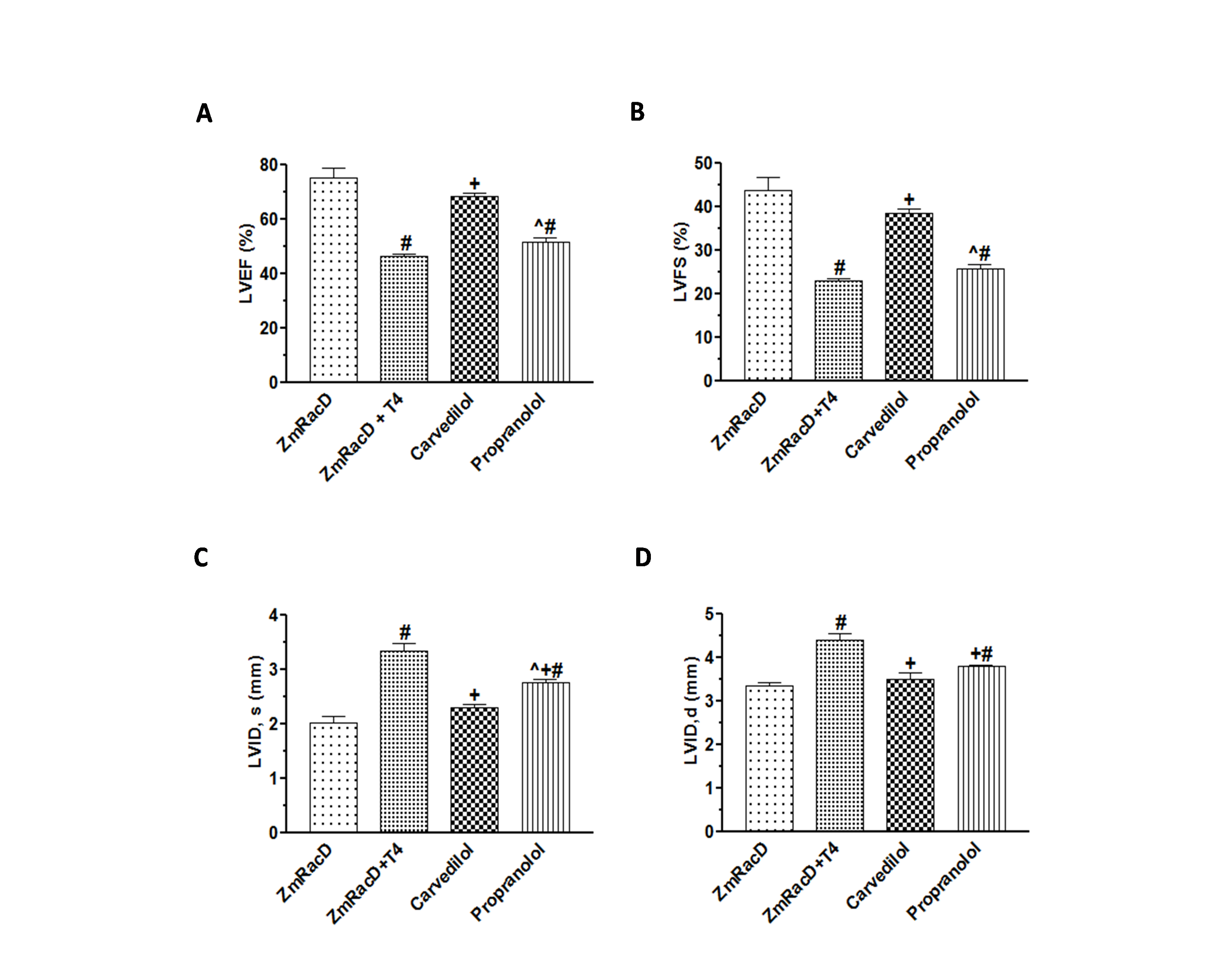

Supplement: Figure S2 — Comparative Effects of Carvedilol and Propranolol on the Left Ventricle (LV) Function and Internal Diameters of T4-treated ZmRacD Mice. Representative bar graphs for LV ejection fraction (EF) (A), fractional shortening (FS) (B), internal diameter during systole (LVID, s) (C), and internal diameter during diastole (LVID, d) (D). # is significant change compared to untreated ZmRacD mice,+ is significant change compared to T4-treated ZmRacD mice and ∧ is significant change compared to T4-supplemented transgenic mice that pre-treated with carvedilol. (TIF) [file pone.0042500.s002.tif]
